# Cefiderocol in Difficult-to-Treat Nf-GNB in ICU Settings

**DOI:** 10.1186/s13613-024-01308-z

**Published:** 2024-05-12

**Authors:** Charles-Hervé Vacheron, Anne Kaas, Jean-Philippe Rasigade, Frederic Aubrun, Laurent Argaud, Baptiste Balanca, Jean-Luc Fellahi, Jean Christophe Richard, Anne-Claire Lukaszewicz, Florent Wallet, Olivier Dauwalder, Arnaud Friggeri

**Affiliations:** 1 Département d’Anesthésie Réanimation, Centre Hospitalier Lyon Sud, Hospices Civils de Lyon, Lyon, France; 2 CIRI-Centre International de Recherche en Infectiologie (Team PHE3ID), Univ Lyon, Université, Claude Bernard Lyon 1, Inserm, U1111, CNRS, UMR5308, ENS Lyon, 46 allée d’Italie, Lyon, 69007 France; 3 Institut des Agents Infectieux, Hospices Civils de Lyon, Lyon, France; 4 Département d’Anesthésie Réanimation douleur, Groupe Hospitalier Nord, Hospices Civils de Lyon, Lyon, France; 5 Research on Health Performance (RESHAPE), INSERM U1290, Université Claude Bernard Lyon 1, Lyon, France; 6 Service de Médecine Intensive-Réanimation, Hôpital Edouard Herriot, Hospices Civils de Lyon, Lyon, France; 7 Faculté de Médecine Lyon-Est, Université Claude Bernard Lyon 1, Université de Lyon, Lyon, France; 8 Hospices Civils de Lyon Centre de recherche en neurosciences de Lyon, Lyon, France; 9 Service d’Anesthésie-Réanimation, Hôpital Universitaire Louis Pradel, Hospices Civils de Lyon, Lyon, France; 10 Laboratoire CARMEN, Inserm U1060, Université Claude Bernard Lyon 1, Lyon, France; 11 CREATIS INSERM, 1044 CNRS 5220 Villeurbanne, France; 12 Médecine Intensive Réanimation-Hôpital de la Croix-Rousse, Lyon, France; 13 CHU Hôpital Edouard Herriot Pathophysiology of Injury-Induced Immunosuppression, Université Lyon 1, Lyon, France; 14 Département d’Anesthésie Réanimation, Centre Hospitalier Lyon Sud Hospices Civils de Lyon, Pierre-Bénite, France; 15 Hospices Civils de Lyon, 24/7 Microbiology Platform, Institut des Agents Infectieux, Centre de Biologie et Pathologie Nord, Lyon, France; 16 Centre Hospitalier Lyon Sud Hospices Civils de Lyon, Service d’anesthésie- réanimation, 165 chemin du Grand Revoyet, 69495 Pierre Benite, France

**Keywords:** Cefiderocol, ICU, Relapse, Clinical cure

## Abstract

**Background:**

The efficacy and safety of cefiderocol in ICU patients with difficult-to-treat resistance (DTR) non-fermenting Gram-negative bacteria (Nf-GNB) are not as well-established. Consequently, we conducted a cohort study to compare Cefiderocol with the Best Available Therapy (BAT) in ICU patients.

**Methods:**

We included adult patients from 9 different ICUs, including a burn ICU unit, from 2019 to 2023 treated with Cefiderocol for DTR Nf-GNB isolated from the blood or lungs. We matched each patient at a 1:2 ratio based on the same DTR Nf-GBN isolated pathogen, and when possible, within the same type of ICU (burn unit or not). The primary endpoint of the study was the clinical cure at 15 days, with secondary endpoints including clinical cure at 30 days, relapse, and in-ICU mortality. For each outcome, adjusted odds ratios were estimated using bidirectional stepwise regression in a final model, which included 13 preselected confounders.

**Results:**

We included 27 patients with cefiderocol, matched with 54 patients receiving the BAT. Four patients were not exactly matched on the type of ICU unit. Characteristics were comparable between groups, mostly male with a Charlson Comorbidity Index of 3 [1–5], and 28% had immunosuppression. Cefiderocol patients were most likely to have higher number of antibiotic lines. The main DTR Nf-GNB identified was *Pseudomonas aeruginosa* (81.5%), followed by *Acinetobater baumanii* (14.8%) and *Stenotrophomonas maltophilia* (3.7%). Pneumonia was the identified infection in 21 (78.8%) patients in the Cefiderocol group and in 51 (94.4%) patients in the BAT group (*p* = 0.054). Clinical cure at 15 and 30-day and the in-ICU mortality was comparable between groups, however relapse was higher in the cefiderocol group (8-29.6% vs. 4-7.4%;aOR 10.06[1.96;51.53])

**Conclusion:**

Cefiderocol did not show an improvement in clinical cure or mortality rates compared to BAT in the treatment of DTR Nf-GNB, but it was associated with a higher relapse rate.

**Supplementary Information:**

The online version contains supplementary material available at 10.1186/s13613-024-01308-z.

## Key points

*What was known*:

•The use of cefiderocol remains debated and under-evaluated in patients admitted to the ICU.

*What this article tells us*:

•In a cohort of 27 patient with cefiderocol treatment, compared with 54 patients treated with best available therapy, no difference was observed compared regarding the 15 and 30-day clinical cure, and in ICU mortality.

•However, a higher risk of relapse was observed in the cefiderocol group: 29% vs. 7%.

**Supplementary Information:**

The online version contains supplementary material available at 10.1186/s13613-024-01308-z.

## Introduction

Cefiderocol is a new siderophore cephalosporin approved by the FDA in 2019 [1, 2]. With a broad spectrum of activity against carbapenemase-producing Gram-negative bacteria, cefiderocol is the first siderophore antibiotic to reach late-stage development and FDA approval, showing good patient tolerance [3].

Non-fermenting Gram-negative bacteria (Nf-GNB), particularly *Pseudomonas aeruginosa*, are a significant burden in intensive care units. Moreover, these pathogens are at high risk of developing resistance, potentially becoming difficult-to-treat resistant (DTR) organisms, especially in the ICU setting. These bacteria are associated with increased mortality, longer ICU stays, and display a wide disparity in prevalence across Europe. In 2022, the rates of Drug-Resistant (DTR) invasive isolates of Pseudomonas *spp.* and Carbapenem-resistant *Acinetobacter baumannii* (CRAB) reached up to 13% and 36%, respectively, as highlighted by the European Centre for Disease Prevention and Control (ECDC) [4].

The current knowledge about cefiderocol is primarily based on randomized controlled trials that compared to carbapenems, which therefore do not specifically address DTR Nf-GNB, or Best Available Therapy (BAT). For instance, in the CREDIBLE-CR trial, cefiderocol was compared to BAT (mainly colistin-based) in 152 patients with DTR GNB infections. This trial demonstrated a higher rate of microbiological eradication and a lower risk of relapse in the cefiderocol arm, although it did not prove a higher rate of clinical success at the time of outcome assessment. Surprisingly, mortality was higher in the cefiderocol arm at both 14 and 28 days, and notably, the study population was not limited to patients admitted to intensive care units. The APEKS-NP trial, focusing on nosocomial pneumonia, involved 292 participants and demonstrated the non-inferiority of cefiderocol in terms of clinical cure, microbiological eradication, and mortality compared to meropenem. However, none of these trials were restricted to ICU patients, and only the CREDIBLE-CR trial focused on carbapenem-resistant pathogen. Some studies suggest that cefiderocol could be a therapeutic alternative to the current treatments available for managing infections caused by multi-drug resistant (MDR) bacteria in the ICU care setting [5, 6]. Therefore, we conducted a retrospective cohort study to evaluate the efficacy and safety of cefiderocol compared to Best Available Therapy in the ICU setting for treating difficult-to-treat Nf-GNB.

## Materials and methods

### Study setting

This retrospective, multicentric study was conducted across nine different university intensive care units, including a burn unit. The study adhered to the ethical standards established in the 1964 Declaration of Helsinki and its subsequent amendments. The study received approval from the Institutional Review Board (CSE-HCL – IRB 00013204-22_547).

### Eligibility criteria

We included adult patients (18 years or older) admitted to the ICU between January 1, 2019, and June 1, 2023, who were treated with Cefiderocol for a difficult-to-treat (DTR) infection caused by non-fermenting Gram-negative bacteria (Nf-GBN) with an identified infection site in the blood (bloodstream infection) or the lungs (pneumonia). Patients with infections caused by more than one DTR Nf-GBN or with a duration of treatment of less than 48 h were excluded from the study.

The chosen definition of DTR Nf-GNB was established for *Pseudomonas aeruginosa* and *Acinetobacter baumanii* as resistance to all fluoroquinolones and all β-lactam categories (except for Cefiderocol, Ceftazidime avibactam, Ceftolozane/tazobactam), including carbapenems. For *Stenotrophomonas maltophilia*, resistance to Trimethoprim/sulfamethoxazole was also a requirement [7].

Afterward, a matching process was carried out for every germ, at a 1:2 ratio, pairing them with another ICU patient exhibiting identical microorganism, DTR profile, and ideally, being situated in the same type of ICU (either a standard ICU or a burn unit ICU).

### Data sources

The data about the Nf-GBN were obtained from the microbiological laboratory database (*Clinisys GLIMS* ®, Glasgow, Scotland), and the electronic health record (*IntelliSpace Critical Care and Anesthesia (ICCA)* ®, Philips, Amsterdam, Netherlands). The electronic health record then provided the following variables: Socio-demographic data and baseline characteristics: Age, sex, Charlson Comorbidity Index, organ transplantation, type of ICU admission (Medical, Surgical, or Burn), Immunosuppression (Immunosuppression (based on a patient getting an immuosuppressive treatment (chemotherapy, radiation, long term or recent high dosesteroids) or having an immuosuppressive condition (e.g. leukemia, lymphoma, multiple myeloma, AIDS), or with a HLA-DR dose < 8000 antibodies bound per cell [8, 9]), number of antibiotic lines before the infection (antibiotic line defined as the number of antibiotic/ association of antibiotic previously used during the intensive care hospitalization), and organ failure on the day of the infection, defined as follows:

- Hemodynamic failure: requirement of vasoactive amines during the last 24 h of the infection.
- Renal: Continuous Renal Replacement Therapy or Acute Kidney Injury KDIGO III.
- Respiratory: Requirement of mechanical ventilation.
- Neurologic: GCS < 9 without sedative drugs.
- Characteristics of the infection: Days from admission to infection, localization, associated pathogen isolated, antibiotic used. The administration of the cefiderocol was performed intermittently at 2 g per 8 h, and the dosage was adapted to the renal clearance.
- Adverse events attributable to cefiderocol or BAT: diarrhea, candidiasis, skin rash, cytolysis, *Clostridioides difficile* infection.
- Follow-up of the infection: clinical cure at 15-day and 30-day (defined by the absence of antimicrobial treatment at 15 days for the same infection site, and the absence of clinical or biological signs of infection), relapse (defined as another infection after clinical cure caused by the same DTR Nf-GNB), duration of mechanical ventilation, delay from infection to ICU discharge, length of ICU stay, and in-ICU mortality.

### Outcome

Our primary endpoint was the clinical cure 15 days after the infection. The secondary endpoints included clinical cure at 30 days, relapse, and in-ICU mortality.

### Statistical methods

Descriptive statistics were expressed by the median and interquartile range [IQR] for quantitative variables and by the number and percentage (%) for qualitative variables. Differences between groups were estimated using the Wilcoxon rank-sum test for quantitative variables, and the Chi-squared test for qualitative variables or Fisher’s exact test when applicable.

To estimate the impact of the use of cefiderocol on each of the outcomes, a full model was built with 15 confounder selected *a priori* : sex, age, SAPS II, immunosuppression, transplantation, Charlson comorbidity index, type of ICU admission (medical, surgical, or burn), presence of hemodynamic, renal, respiratory and neurologic organ failure at the time of infection, pathogen of the infection, previous use of new β-lactam/β-lactamase inhibitor (Ceftazidime avibactam or Ceftolozane/tazobactam), presence of a polymicrobial infection and delay between ICU admission and occurrence of the infection. Then, a bidirectional Stepwise Algorithm was used to select the final model using the best Akaike information criterion. The treatment group was forced into the model. The final impact of the cefiderocol treatment was expressed using adjusted Odds Ratio associated with their 95% confidence interval [95%CI]. As a sensitivity analysis, the Relapse was also studied using competing risk regression, with death or discharge of ICU treated as competing event. The same confounder and variable selection process was performed. The statistical threshold was set at 0.05. Analyses were performed using the R software v3.4.3 [10].

## Results

**Fig. 1:**
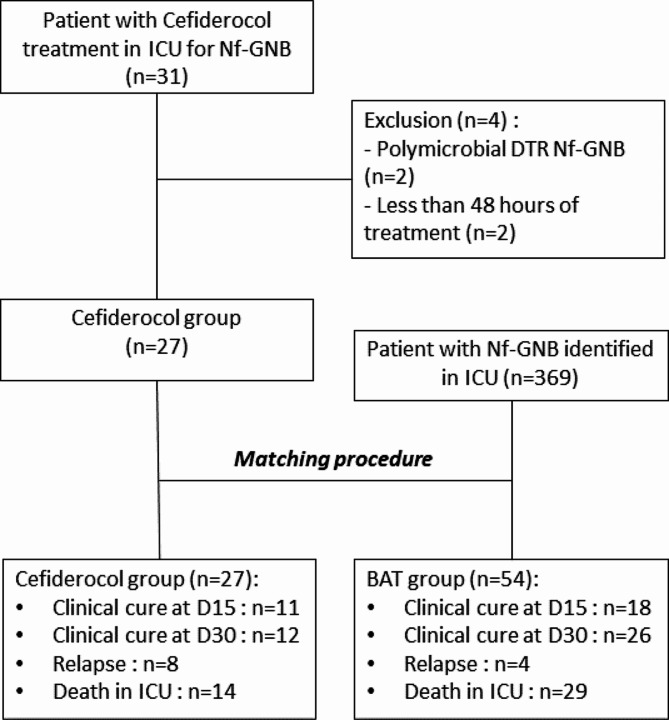
Flow chart Nf-GNB : Non fermenting Gram-Negative Bacteria; BAT : Best available treatment

Out of the 31 patients treated with cefiderocol for DTR Nf-GNB, 2 patients with more than one DTR Nf-GBN and 2 patients with less than 48 h of treatment were excluded, leaving a final total of 27 patients in the cefiderocol group (Fig. 1). We applied an exact matching procedure based on the identical pathogen, the same DTR profile, and if possible, the same type of ICU for 27 patients, resulting in a comparison group of 54 patients receiving the Best Available Therapy (BAT). Only 4 matched patients could not be precisely matched based on the ICU profile; as a result, 4 patients in the cefiderocol group from the burn unit were matched with 4 patients in a conventional ICU unit.

### Description of the population (table 1)

**Table 1:** Patient characteristics

Variable	Cefiderocol group *n* = 27	BAT group *n* = 54	*p* value
Sexe, Male	20 (74.1%)	35 (64.8%)	0.556
Age, year	58 [42–65]	60 [45–67]	0.968
Charlson Comorbidity Index	4 [1–5]	3 [1–5]	0.781
Immunosupression	11 (40.7%)	12 (22.2%)	0.139
Organ transplantation	5 (18.5%)	10 (18.5%)	1.000
*Lung*	1	8	
*Heart*	1	2	
*Liver*	1	0	
*Liver-Kidney*	1	0	
*Bone Marrow*	1	0	
Type of ICU admission			0.341
*Medical*	13 (48.1%)	17 (31.5%)	
*Surgical*	4 (14.8%)	11 (20.4%)	
*Burn*	10 (37.0%)	17 (31.5%)	0.222
Previous antibiotic line			0.010
*0*	0 (0.0%)	0 (0.0%)	
*1*	2 (7.4%)	15 (27.8%)	
*2*	10 (37.0%)	26 (48.1%)	
*≥ 3*	15 (55.6%)	13 (24.1%)	
Previous use of new β-lactam/β-lactamase inhibitor	10 (37.0%)	6 (11.1%)	0.014
SAPS II score	55 [42–66]	47 [43–66]	0.217
Delay from admission to infection, days	29 [16–54]	25 [10–59]	0.346
Organ failure at the occurrence of infection			
*Hemodynamic failure*	21 (77.8%)	43 (79.6%)	1.000
*Renal Failure*	13 (48.1%)	28 (51.9%)	0.937
*Respiratory failure*	25 (92.6%)	53 (98.1%)	0.256
*Neurologic failure*	9 (33.3%)	22 (40.7%)	0.686

The patients included were predominantly male (55–67.9%), with an average age of 58 [44–67] years old and were mostly admitted for medical reasons, followed by burn and surgical admission. They had a Charlson Comorbidity Index of 3 [1–5] and 28.4% (23 patients) presented with immunosuppression. No statistical differences were observed between the groups. During their ICU stay, 25 (92.6%) patients in the Cefiderocol group and 53 (98.1%) in the BAT group required mechanical ventilation (*p* = 0.597).

Before the administration of the antibiotic, none of the patients were antibiotic naive. The Cefiderocol group was more likely to have a higher number of previous antibiotic (15 (55.6%) patients with ≥ 3 lines of antibiotic treatment) compared to the BAT group (13 (24.1%) patients with ≥ 3 lines of antibiotic). Details of the previous antimicrobial therapies used in the ICU can be found in Supplementary Table 1. Notably, the cefiderocol group had a higher rate of previous use of previous use of new β-lactam/β-lactamase inhibitor (10 (37.0%) vs. 6 (11.1%)). Organ failure at the time of infection was comparable, primarily involving respiratory and hemodynamic failure.

### Characteristics of the infection (table 2)

**Table 2:** Characteristic of the infection

Variable	Cefiderocol group *n* = 27	BAT group *n* = 54	*p* value
Pathogen identified			
*Pseudomonas aeruginosa*	22 (81.5%)	44 (81.5%)	
*Acinetobacter baumannii*	4 (14.8%)	8 (14.8%)	
*Stenotrophomonas maltophilia*	1 (3.7%)	2 (3.7%)	
Localization of the infection			0.054
*Pneumonia*	21 (77.8%)	51 (94.4%)	
*Bloodstream infection*	6 (22.2%)	3 (5.6%)	
Polymicrobial infection	22 (81.5%)	36 (66.7%)	0.257
Number of associated pathogen			0.147
1	13 (59.1%)	27 (75.0%)	
2	5 (22.7%)	8 (22.2%)	
*≥*3	4 (18.2%)	1 (2.8%)	
Antibiotic therapy			
*Cefiderocol*	27 (100%)	0 (0%)	
*Ceftazidime avibactam*	0 (0%)	28 (51.9%)	
*Ceftolozane/tazobactam*	0 (0.0%)	8 (14.8%)	
*Carbapenem*	0 (0.0%)	2 (3.7%)	
*Imipenem/cilastatin/relebactam*	0 (0.0%)	2 (3.7%)	
*Aztreonam*	0 (0.0%)	2 (3.7%)	
*Trimethoprim/sulfamethoxazole*	0 (0.0%)	1 (1.9%)	
*Fluoroquinolone*	0 (0.0%)	6 (11.1%)	
*Colistin*			
Intravenous	1 (3.7%)	14 (25.9%)	
Inhaled	6 (22.2%)	16 (29.6%)	
Duration of antimicrobial therapy	13 [8–15]	14 [12–15]	0.241

The predominant DTR Nf-GNB identified was *Pseudomonas aeruginosa* (81.5%), followed by *Acinetobacter baumannii* (14.8%) and *Stenotrophomonas maltophilia* (3.7%). Pneumonia was the identified infection in 21 (78.8%) patients in the Cefiderocol group and in 51 (94.4%) patients in the BAT group (*p* = 0.054). An additional microorganism was present in 22 (81.5%) patients in the Cefiderocol group and 36 (66.7%) patients in the BAT group (*p* = 0.257). Further details regarding the associated microorganisms can be found in Supplementary Table 2. The duration of antibiotic was 13 [8–16] days in the Cefiderocol group and 14 [12–15] days in the BAT group (*p* = 0.241). In the Cefiderocol group, 7 patients also received Colistin treatment, predominantly inhaled Colistin. The Cefiderocol was adapted to the renal clearance in 20 (74.1%) of the patient. In the BAT group, the most commonly used antibiotic was Ceftazidime-avibactam (28 patients, 51.9%), followed by Colistin and Ceftolozane/tazobactam. In the Cefiderocol group, we noticed a higher associated rate of resistance to new β-lactam (Supplementary Table 3).

### Outcome of the patients (table 3)

**Table 3:** Outcomes of the patients

Variable	Cefiderocol group *n* = 27	BAT group *n* = 54	*p* value
Clinical cure at 15-day	11 (40.7%)	18 (33.3%)	0.682
Clinical cure at 30-day	12 (44.4%)	26 (48.1%)	0.937
Relapse	8 (29.6%)	4 (7.4%)	0.017
Delay infection-relapse, days	43 [35–46]	22 [15–31]	0.106
Duration of mechanical ventilation, days	93 [46–122]	59 [30–75]	0.050
Delay from infection to ICU discharge, days	47 [16–77]	29 [14–44]	0.090
Length of ICU stay, days	95 [47–134]	65 [36–97]	0.087
In ICU mortality	14 (51.9%)	29 (53.7%)	1.000

**Fig. 2:**
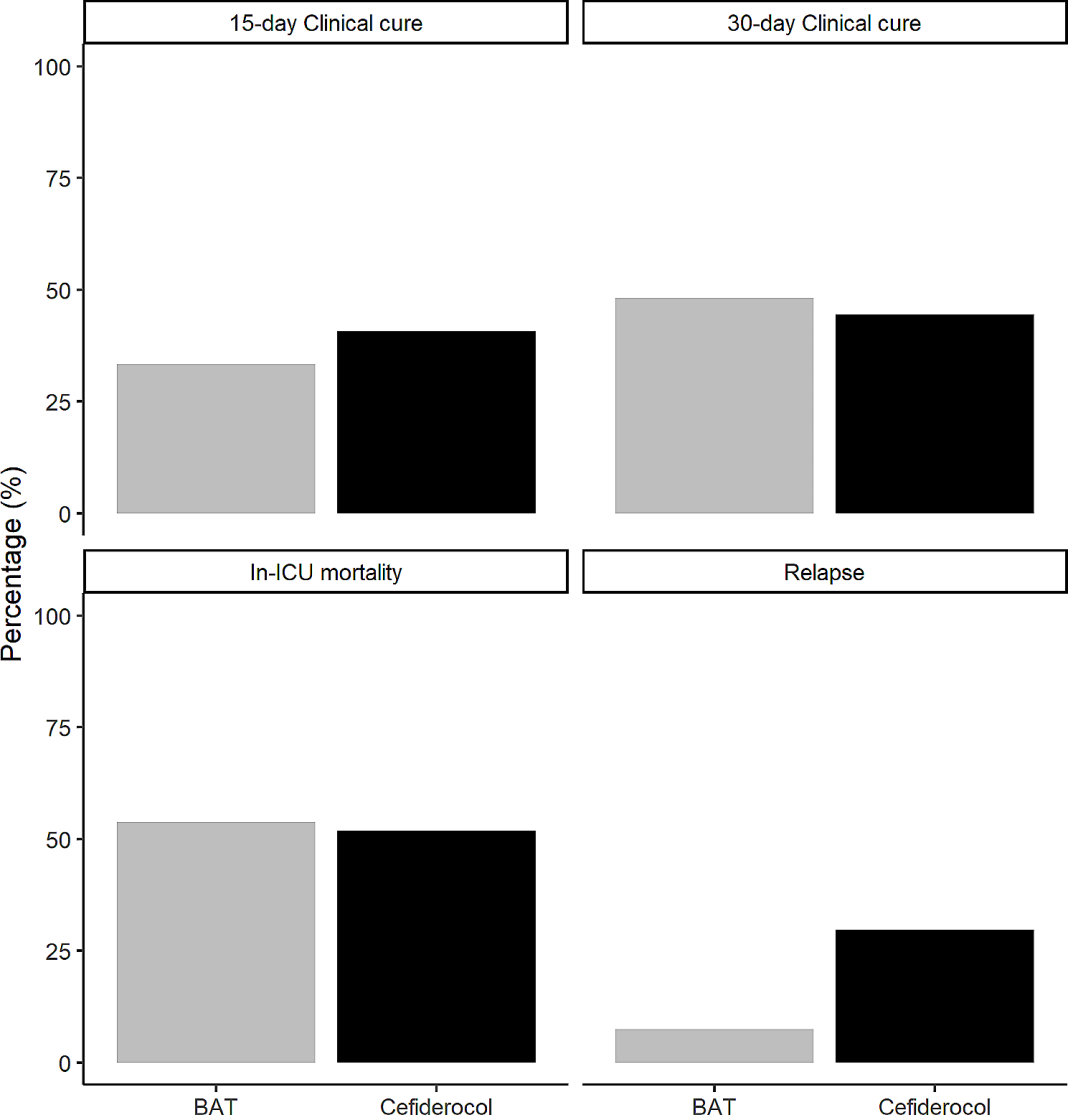
Description of the clinical cure, relapse and clinical cure

The clinical cure at 15 days was similar between groups, with 40.7% (11 patients) in the cefiderocol group and 33.3% (18 patients) in the BAT group achieving cure (*p* = 0.682). However, the rate of relapse was higher in the cefiderocol group, with 8 (29.6%) patients experiencing relapse compared to 4 (7.4%) patients in the BAT group (*p* = 0.017) Figure 2. No emergence of resistance of cefiderocol was noted in the 8 relapses of the cefiderocol group, and cefiderocol was used to treat this relapse in 7 of the 8 relapses. In-ICU mortality, duration of mechanical ventilation, and time from infection to ICU discharge were comparable between the groups. The incidence of adverse events was similar between the cefiderocol and BAT groups, as documented in Supplementary Table 4.

These comparisons across different endpoints were confirmed after adjusting for main confounding variables, particularly regarding the higher risk of relapse, with an adjusted odds ratio (aOR) of 10.06 [1.96;51.53], *p* = 0.005 (Table 4; Supplementary Table 5). These results were confirmed in the sensitivity analysis (sub-Hazard Ratio 8.38 [1.91;36.67]; Supplementary Table 6).

**Table 4:** Impact of the Cefiderocol on the different endpoints

Variable	Adjusted Odds Ratio	*p* value
Clinical cure at 15-day	1.65 [0.54;5.08]	0.382
Clinical cure at 30-day	0.75 [0.26;2.19]	0.597
Relapse	10.06 [1.96;51.53]	0.005
In ICU mortality	1.11 [0.37;3.35]	0.855

## Discussion

The use of cefiderocol in the ICU is currently under-evaluated in the intensive care setting. The findings of this present study suggest that the use of cefiderocol, compared to the BAT, does not differ significantly in clinical cure or in-ICU mortality, but it does suggest a higher rate of infection relapse.

Among randomized controlled trials conducted to evaluate the efficacy of cefiderocol, three are noteworthy. The first was a comparison of cefiderocol versus imipenem-cilastatin for treating complicated urinary tract infections caused by Gram-negative uropathogens. This phase 2 trial included 452 patients, primarily with infections caused by Enterobacterales, and observed a higher clinical success rate in the cefiderocol arm [2]. The CREDIBLE-CR trial randomized 152 patients to receive either cefiderocol (101 patients) or the BAT (51 patients) [11]. These patients had DTR-GNB documented pneumonia, bloodstream infection, or sepsis, in various settings (ICU or not). The trial reported similar clinical success rates at the end of treatment in both groups (66% versus 58%) but noted a higher mortality rate in the cefiderocol arm at 14, 28 days, and at the end of the study. Lastly, the APEKS-NP trial included 145 participants in the cefiderocol arm and 152 in the meropenem arm, all suffering from pneumonia with Multi Drug Resistant GNB, most of which were Enterobacterales [12]. This trial showed comparable 14-day mortality rates and clinical cures. However, none of these trials specifically focused on DTR Nf-GNB or on the unique conditions of the ICU-care setting.

Therefore, the retrospective cohort study by Russo et al. specifically focused on Carbapenem-Resistant *Acinetobacter baumannii* in COVID-19 associated Ventilator-Associated Pneumonia (VAP) [13]. The study, which involved 73 patients with VAP, compared Cefiderocol-containing regimens with Colistin-containing regimens. The results showed that Cefiderocol was associated with better survival (HR 0.44 [0.22;0.66]). Despite the well-known impact on mortality of COVID-associated Ventilator-Associated Pneumonia (VAP), and its high incidence which could potentially explain the significant impact of treatment on mortality rates, the patients on the Colistin regimen had a surprisingly high 30-day mortality rate (98.1%) and only 19 patients in this study were treated with Cefiderocol [14, 15].

These results are inconsistent with other studies, such as the cohort from Mazzitelli, study involving 111 patients with Carbapenem-Resistant *Acinetobacter baumannii* infections [16]. This study showed a higher, yet not statistically significant, mortality rate in the cefiderocol group compared to the colistin group (51% vs. 37%, *p* = 0.130). These results were confirmed in several study and meta-analysis, which reported better outcomes in the specific setting of Carbapenem-Resistant *Acinetobacter baumannii* infections with cefiderocol [17–20].

In another instance, Wicky et al. reported on 16 ICU patients treated with cefiderocol for DTR Nf-GNB. In this descriptive study, they observed a clinical cure rate of 68.7% and an ICU mortality rate of 31.3%. Notably, persistent colonization was found in 81.3% of the patients, and a relapse was documented in 56.3% of them. The duration of antibiotic therapy was short, averaging 8 [7-13.5] days, and 31.3% of the patients received a combination of antibiotics, mainly with colistin [21].

A possible reason for the high relapse rate could be that more patients in the cefiderocol group had used new β-lactam/β-lactamase inhibitor before, which might indicate that cefiderocol was given as a rescue therapy in 37% of the patients (compared to 11% in the BAT group). Even though many patients in the cefiderocol group had not used new β-lactam/β-lactamase inhibitor before, and that this prior use was not finally associated with this outcome, this remains a potential explanation for the high relapse rate. Another observation is the use of cefiderocol in a context of higher resistance.

There are also specific concerns about the emergence of resistance during treatment with cefiderocol. As described by some authors, doubts exist regarding its efficacy against non-fermenting GNB, with several resistance mechanisms identified for cefiderocol [24, 25]. Moreover, difficulties for cefiderocol laboratory testing were also reported by EUCAST, choosing disc diffusion method for resistance screening as performed in our study [26]. Additionally, questions remain about whether to use cefiderocol as monotherapy or in combination with other antibiotics, but currently, there is no clinical data available to support either practice [27].

We observed a notably high percentage of polymicrobial infections : the successive exposure to antimicrobial agents across multiple infection episodes favors the emergence of highly resistant strains and the coexistence of multiple pathogens within the host – 70% in our cohort. Furthermore, the prolonged ICU stays preceding these infections further exacerbate the likelihood of polymicrobial involvement, and the ICU-acquired immunosuppression.

Despite the known adverse events attributable to cefiderocol, we confirmed that it remains safe for use in ICU care, likely because the antibiotics administered in the BAT group had more severe adverse events. Indeed, the toxicity of cefiderocol has been extensively assessed in randomized controlled trials and preclinical studies [28, 29]. It has the advantage of probably presenting no clinically significant drug-drug interaction (DDI) [30].

However, this study is subject to several notable limitations that must be acknowledged. Firstly, the relatively low number of patients included in the study could potentially limit the generalizability of the findings. Furthermore, there was a lack of matching based on the site of infection. This oversight could result in variations in the treatment outcomes, as the efficacy of cefiderocol may differ depending on the infection site, a factor not accounted for in our study design. Additionally, the retrospective nature of the study introduces inherent biases, as it relies on pre-existing data and lacks the stringent controls of prospective research: for example, the ‘clinical cure’ was not assessed in a blinded manner, not standardized – based on the medical note, physiological and biological parameters at the time of the evaluation - and is therefore not a objective endpoint such as mortality. Finally, on the matched DTR Nf-GNB, we observed a higher proportion of resistance, especially to other new β-lactam. This constatation, associated to the higher number of previous antimicrobial line might explain the higher rate of relapse rate in the cefiderocol group. Therefore, these results need should be confirmed through larger real-life data cohorts. Moreover, specific randomized controlled trials are necessary to validate our observations and ensure that the conclusions drawn are robust and applicable to broader clinical practices.

## Conclusion

In conclusion, our study provides important insights into the efficacy and safety of cefiderocol in the treatment of DTR Nf-GNB in ICU settings. We observed that while cefiderocol is comparable to the best available therapy (BAT) in terms of clinical cure and in-ICU mortality, it may be associated with a higher rate of infection relapse.

### Electronic supplementary material

Below is the link to the electronic supplementary material.

Supplementary Material 1

## Data Availability

Upon reasonable request.
