# Correction: Cefiderocol in Difficult-to-Treat Nf-GNB in ICU Settings

**DOI:** 10.1186/s13613-024-01317-y

**Published:** 2024-05-24

**Authors:** Charles-Hervé Vacheron, Anne Kaas, Jean-Philippe Rasigade, Frederic Aubrun, Laurent Argaud, Baptiste Balanca, Jean-Luc Fellahi, Jean Christophe Richard, Anne-Claire Lukaszewicz, Florent Wallet, Olivier Dauwalder, Arnaud Friggeri

**Affiliations:** 1 Département d’Anesthésie Réanimation, Centre Hospitalier Lyon Sud, Hospices Civils de Lyon, Lyon, France; 2 CIRI-Centre International de Recherche en Infectiologie (Team PHE3ID), Univ Lyon, Université, Claude Bernard Lyon 1, Inserm, U1111, CNRS, UMR5308, ENS Lyon, 46 allée d’Italie, Lyon, 69007 France; 3 Institut des Agents Infectieux, Hospices Civils de Lyon, Lyon, France; 4 Département d’Anesthésie Réanimation douleur, Groupe Hospitalier Nord, Hospices Civils de Lyon, Lyon, France; 5 Research on Health Performance (RESHAPE), INSERM U1290, Université Claude Bernard Lyon 1, Lyon, France; 6 Service de Médecine Intensive-Réanimation, Hôpital Edouard Herriot, Hospices Civils de Lyon, Lyon, France; 7 Faculté de Médecine Lyon-Est, Université Claude Bernard Lyon 1, Université de Lyon, Lyon, France; 8 Hospices Civils de Lyon Centre de recherche en neurosciences de Lyon, Lyon, France; 9 Service d’Anesthésie-Réanimation, Hôpital Universitaire Louis Pradel, Hospices Civils de Lyon, Lyon, France; 10 Laboratoire CARMEN, Inserm U1060, Université Claude Bernard Lyon 1, Lyon, France; 11 CREATIS INSERM, 1044 CNRS 5220 Villeurbanne, France; 12 Médecine Intensive Réanimation-Hôpital de la Croix-Rousse, Lyon, France; 13 CHU Hôpital Edouard Herriot Pathophysiology of Injury-Induced Immunosuppression, Université Lyon 1, Lyon, France; 14 Département d’Anesthésie Réanimation, Centre Hospitalier Lyon Sud Hospices Civils de Lyon, Pierre-Bénite, France; 15 Hospices Civils de Lyon, 24/7 Microbiology Platform, Institut des Agents Infectieux, Centre de Biologie et Pathologie Nord, Lyon, France; 16 Centre Hospitalier Lyon Sud Hospices Civils de Lyon, Service d’anesthésie- réanimation, 165 chemin du Grand Revoyet, Pierre Benite, 69495 France

**Correction: Annals of Intensive Care 2024 :14 (73)**

10.1186/s13613-024-01308-z

The wrong Supplementary file (2) was originally published with this article; it has now been replaced with the correct file.

The original article has been corrected.

