# Supplementary material for: Cefiderocol in Difficult-to-Treat Nf-GNB in ICU Settings
Source: Ann Intensive Care. 2024 May 12;14:73. doi: 10.1186/s13613-024-01308-z (PMC11089025; doi:10.1186/s13613-024-01308-z)
Supplement: Supplementary file 1 — Supplementary Material 1 [file 13613_2024_1308_MOESM1_ESM.docx]

**Supplementary Table 1 : Previous antibiotic used**

| Variable | Cefiderocol group  n=27 | BAT group  n=54 | *p value* |
| --- | --- | --- | --- |
| 3^rd^ generation Cephalosporin | 19 (70.4%) | 12 (22.2%) | 0.649 |
| Piperacillin Tazobactam | 12 (44.4%) | 26 (48.1%) | 0.937 |
| Carbapenem | 17 (63.0%) | 22 (40.7%) | 0.099 |
| Colistin | 7 (25.9%) | 2 (3.7%) | 0.005 |
| Ceftazidime | 1 (3.7%) | 1 (1.9%) | 1.000 |
| Ceftazidime avibactam | 8 (29.6%) | 3 (5.6%) | 0.005 |
| Ceftolozane/ tazobactam | 2 (7.4%) | 3 (5.6%) | 1.000 |
| Aminoglycoside | 8 (29.6%) | 4 (7.4%) | 0.017 |
| Metronidazole | 5 (18.5%) | 4 (7.4%) | 0.152 |

**Supplementary Table 2 : associated micro-organisms**

|  | Cefiderocol group  n=41 | BAT group  n=50 |
| --- | --- | --- |
| *Achromobacter spp.* | 0 (0.0%) | 1 (2.4%) |
| *Acinetobacter baumanii* | 2 (4.0%) | 1 (2.4%) |
| *Aspergillus spp.* | 0 (0.0%) | 2 (4.9%) |
| *Bacillus cerus* | 1 (2.0%) | 0 (0.0%) |
| *Citrobacter spp.* | 2 (4.0%) | 1 (2.4%) |
| *Enterobacter spp.* | 4 (8.0%) | 1 (2.4%) |
| *Enterococcus faecalis* | 3 (6.0%) | 5 (12.2%) |
| *Enterococcus faecium* | 1 (2.0%) | 3 (7.3%) |
| *Escherichia coli* | 5 (10.0%) | 3 (7.3%) |
| *Klebsiella spp.* | 9 (18.0%) | 2 (4.9%) |
| *Lactobacillus spp.* | 0 (0.0%) | 1 (2.4%) |
| *Moraxella spp.* | 2 (4.0%) | 0 (0.0%) |
| *Morganella spp.* | 4 (8.0%) | 1 (2.4%) |
| *Mucor spp.* | 0 (0.0%) | 1 (2.4%) |
| *Proteus spp.* | 2 (4.0%) | 3 (7.3%) |
| *Pseudomonas spp.* | 4 (8.0%) | 2 (4.9%) |
| *Serratia spp.* | 2 (4.0%) | 1 (2.4%) |
| *Staphylococcus aureus* | 5 (10.0%) | 7 (17.1%) |
| *Staphylococcus non aureus* | 1 (2.0%) | 5 (12.2%) |
| *Stenotrophomonas maltophilia* | 2 (4.0%) | 0 (0.0%) |
| *Streptococcus spp.* | 1 (2.0%) | 0 (0.0%) |
| *Raoultella spp.* | 0 (0.0%) | 1 (2.4%) |

**Supplementary Table 3 : Resistance to new β-lactam**

Cefiderocol resistance (respective Minimal inhibitory concentration (MIC) for the *Pseudomonas aeruginosa* > 2 (mg/L), *Acinetobacter baumannii* > 2 (mg/L), *Stenotrophomonas maltophilia*: > 2 (mg/L).


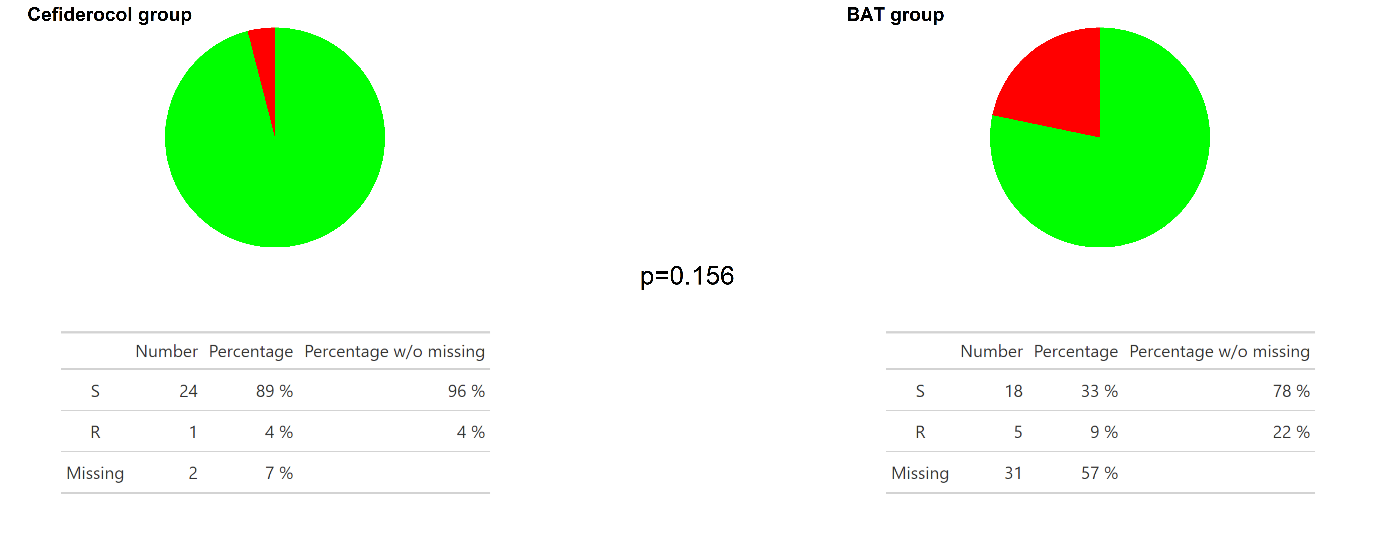


Ceftazidime/Avibactam resistance (respective MIC for the *Pseudomonas aeruginosa* > 8 (mg/L), *Acinetobacter baumannii*  > 8 (mg/L), *Stenotrophomonas maltophilia* > 8 (mg/L).


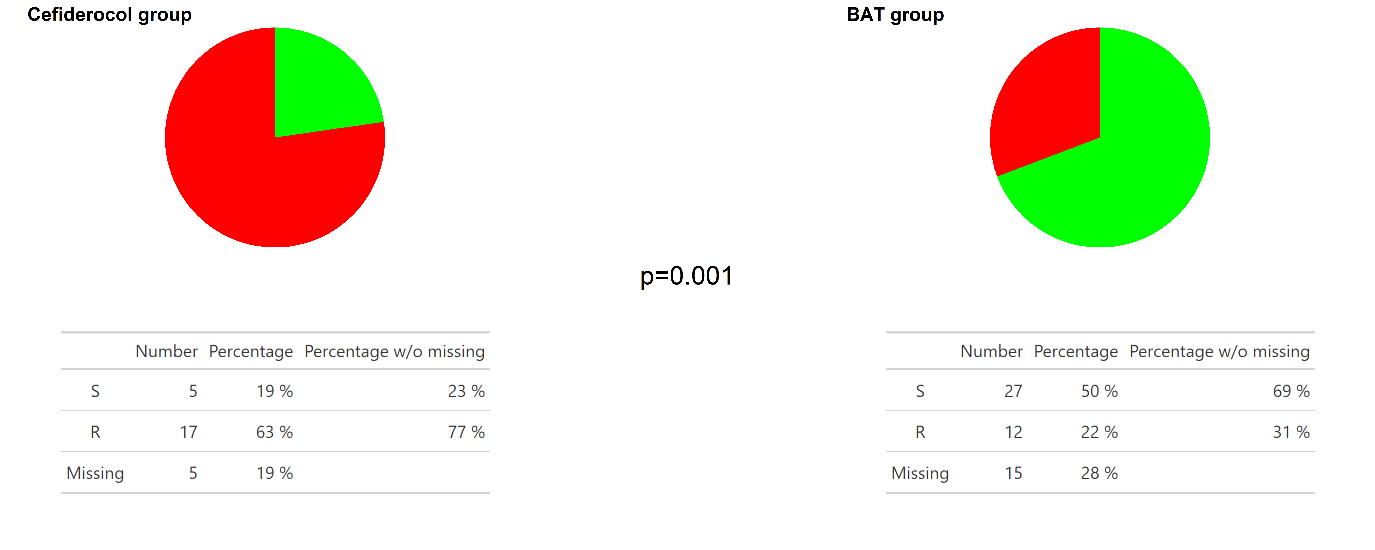


Imipenem/Relebactam resistance (respective MIC for the *Pseudomonas aeruginosa* > 2 (mg/L), *Acinetobacter baumannii*  2 (mg/L), *Stenotrophomonas maltophilia* 2 (mg/L).


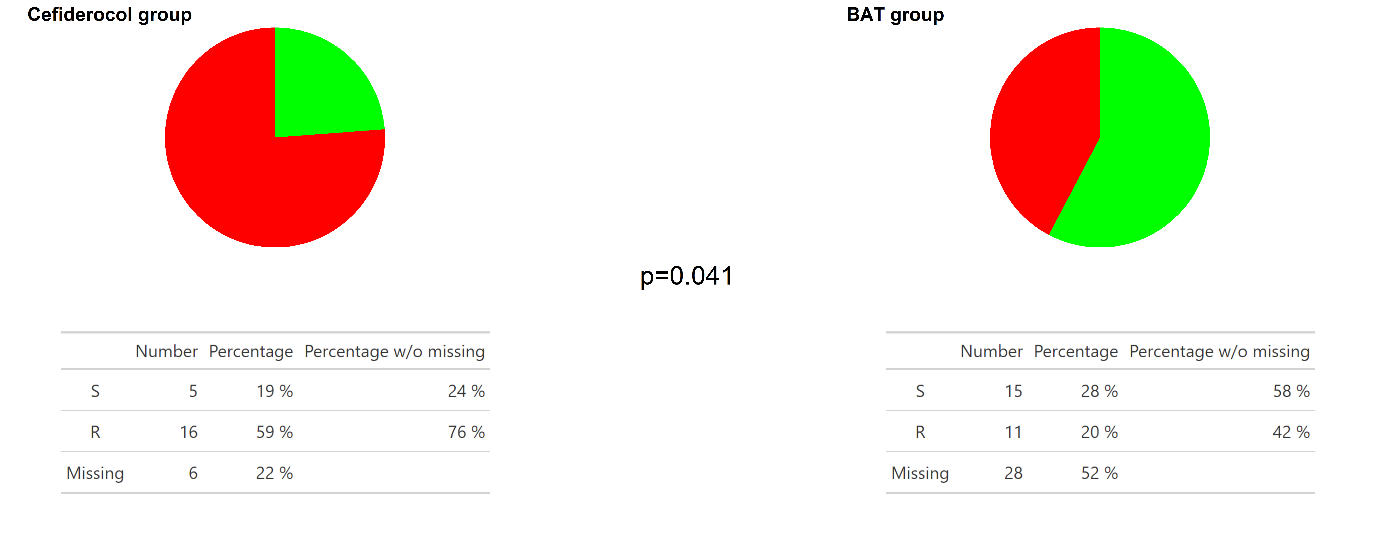


Ceftolozane/Tazobactam resistance (respective MIC for the *Pseudomonas aeruginosa* > 4 (mg/L), *Acinetobacter baumannii* >4 (mg/L), *Stenotrophomonas maltophilia*: >4 (mg/L).


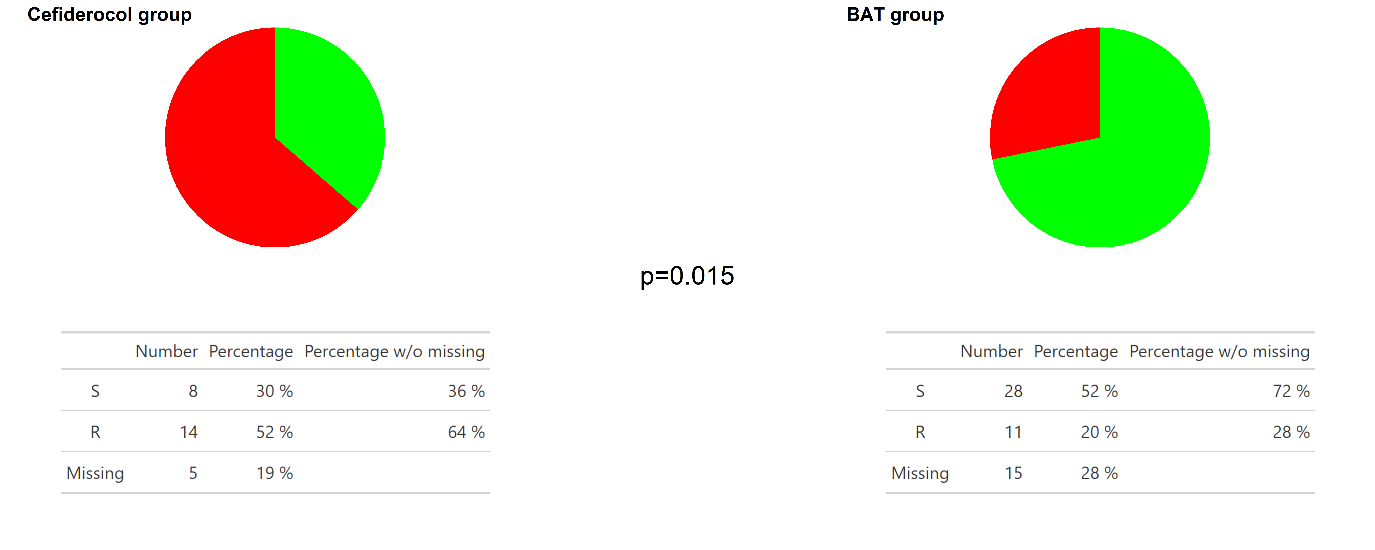


**Supplementary Table 4 : Adverse event**

| Variable | Cefiderocol group  n=27 | BAT group  n=54 | *p value* |
| --- | --- | --- | --- |
| Diarrhea | 3 (11.1%) | 15 (27.8%) | 0.156 |
| Skin rash | 1 (3.7%) | 8 (14.8%) | 0.259 |
| Cytolysis | 2 (7.4%) | 7 (13.0%) | 0.710 |
| Candidiasis | 0 (0.0%) | 3 (5.6%) | 0.547 |
| *Clostridioides difficile* infection | 2 (7.4%) | 7 (13.0%) | 0.710 |

**Supplementary Table 5 : Different model**

Clinical cure at 15-day

| Variable | Adjusted Odds Ratio | *p value* |
| --- | --- | --- |
| Cefiderocol Group | 1.65 [0.54;5.08] | 0.382 |
| Sex, Male | 2.84 [0.88;9.18] | 0.081 |
| Previous use of new β-lactam/β-lactamase inhibitor | 0.24 [0.05;1.09] | 0.065 |
| Hemodynamic failure | 0.21 [0.06;0.75] | 0.017 |
| Delay from admission to infection, per 10 days | 1.09 [0.97;1.23] | 0.136 |

Clinical cure at 30-day

| Variable | Adjusted Odds Ratio | *p value* |
| --- | --- | --- |
| Cefiderocol Group | 0.75 [0.26;2.19] | 0.597 |
| Sex, Male | 6.94 [2.13;22.64] | 0.001 |
| Age, years | 1.06 [1.02;1.11] | 0.005 |
| Charlson Comorbidity index | 0.71 [0.54;0.95] | 0.002 |
| Hemodynamic failure | 0.31 [0.08;1.17] | 0.0838 |

Relapse

| Variable | Adjusted Odds Ratio | *p value* |
| --- | --- | --- |
| Cefiderocol Group | 10.06 [1.96;51.53] | 0.006 |
| Type of admission |  |  |
| Burn | - | - |
| Medical | 4.16 [0.44;39.17] | 0.021 |
| Surgical | 40.06 [2.62;612.05] | 0.007 |
| Renal failure | 0.29 [0.06;1.44] | 0.129 |
| *Pseudomonas* infection | 0.15 [0.02;1.51] | 0.107 |
| Hemodynamic failure | 10.82 [0.75;155.79] | 0.080 |

Sensitivity Analysis : *Competing risk regression*

| Variable | Sub Hazard Ratio | *p value* |
| --- | --- | --- |
| Cefiderocol Group | 8.38 [1.91;36.67] | 0.005 |
| Type of admission |  |  |
| Burn | - | - |
| Medical | 4.22 [0.42;42.67] | 0.223 |
| Surgical | 52.30 [3.38;809.90] | 0.004 |
| Renal failure | 0.29 [0.07;1.11] | 0.069 |
| *Pseudomonas* infection | 0.10 [0.01;1.08] | 0.058 |
| Hemodynamic failure | 7.72 [0.87;68.07] | 0.065 |

In ICU mortality

| Variable | Adjusted Odds Ratio | *p value* |
| --- | --- | --- |
| Cefiderocol Group | 0.99 [0.30;3.22] | 0.985 |
| SAPS II score | 1.04 [1.00;1.08] | 0.040 |
| Sex, Male | 0.14 [0.04;0.49] | 0.002 |
| Charlson Comorbidity index | 1.31 [1.05;1.65] | 0.018 |
| Hemodynamic failure | 5.36 [1.28;22.36] | 0.021 |
